# The impact of visceral obesity on chronic constipation, inflammation, immune function and cognitive function in patients with inflammatory bowel disease

**DOI:** 10.18632/aging.202526

**Published:** 2021-02-17

**Authors:** Yemin Wan, Dan Zhang, Ting Xing, Qiaoling Liu, Yumeng Chi, Huixiang Zhang, Haihua Qian

**Affiliations:** 1 Department of Anorectal Surgery, The Affiliated Hospital of Nanjing University of Chinese Medicine, Nanjing 210029, Jiangsu Province, China

**Keywords:** visceral obesity, inflammatory bowel disease, chronic constipation, inflammation, immune function

## Abstract

Objective: Obesity has gained attention among patients with inflammatory bowel disease (IBD). The impact of visceral obesity on chronic constipation, inflammation, immune function and cognition after diagnosis of IBD is still unknown.

Methods: This is a cross-sectional study of 150 IBD patients. Patients’ visceral adipose tissue (VAT) and subcutaneous adipose tissue (SAT) were measured and were grouped according to visceral obesity. The potential impact of visceral obesity on cognitive function were evaluated using Mini-Mental State Examination. We evaluated patients’ incidence of chronic constipation, levels of interleukin-6 (IL-6), T cells and body mass index in two groups.

Results: The prevalence of visceral obesity was 51% (37 out of 72) for Crohn’s disease (CD) patients and 26% for UC patients (20 out of 78 patients). CD patients with visceral obesity has higher incidence of chronic constipation (81% vs. 57%, *P* = 0.028), higher IL-6 levels (15.28 pg/ml vs. 9.429 pg/ml, *P* = 0.007) and lower CD4^+^ T cells (32.7% vs. 44.0%, *P* < 0.001). VAT/SAT ratio is associated with BMI (*P* < 0.001).

Conclusions: IBD patients had high risks of visceral obesity. CD Patients with visceral obesity had higher prevalence of chronic constipation, higher inflammation levels, decreased immune function.

## INTRODUCTION

Inflammatory bowel disease (IBD) is gastrointestinal disorders that involve chronic inflammation of digestive tract with two types mainly diagnosed: Crohn’s disease (CD) as well as ulcerative colitis (UC) [1]. The abdominal symptoms have been well studied, The abdominal symptoms has been well studied, however the factor that affect IBD patients’ constipation, inflammation, immune function and cognition after several years of IBD disease history have not been comprehensively characterized.

Constipation is a common symptom of IBD, diagnosed by having less than 3 bowel movements per week and / or difficulty in bowel movements. More than 10% IBD patients had chronic constipation [2]. Obesity has gained attention for patients with IBD. Several previous studies have analyzed the association of IBD risk with obesity [3]. An observational study of 524 IBD patients proved that obesity at diagnosis was more common in CD patients versus UC patients (odds ratio 2.02, *P* = 0.0096) [4]. Increasing BMI was found to parallel to risk of CD, rather than UC [4]. Several studies proved that the prevalence of overweight or obesity for CD patients ranged from 40% to 52% in western countries [5, 6]. These studies only evaluated body mass index (BMI) to define obesity and a threshold of obesity was defined as BMI > 30 kg/m^2^. No published articles evaluate further into impact of visceral obesity based on computed tomography (CT) scans on clinical characteristics in the disease process of IBD patients, especially in Chinese population. Thus, we aimed to evaluate the impact of visceral obesity in the remission period of IBD. There is growing interest that the pro-inflammatory cytokine interleukin-6 (IL-6) plays a vital role in the disease process of uncontrolled IBD [7]. It has been proved that IL-6 was increased with active CD [7]. Moreover, IL-6 is a clinical relevant parameter for inflammatory activity of CD and well correlated with relapse of CD during disease remission [7]. Although various cytokines were studied in CD process, IL-6 gained the central pathogenetic role due to its indication of early lesions of new diagnosed patients as well as patients with long history of CD [7, 8]. Actually, IL-6 has a broad effect on immune cells [9]. IL-6 deficiency could lead to impaired both the innate and adaptive immunity [10, 11]. IL-6 receptor (IL-6R) has been proved to be expressed on CD4^+^ T cells [12]. Previous studies have demonstrated that the production of IL-6 and soluble receptors (sIL-6R) released by intestinal macrophages and CD4^+^ T cells in the mucosa of patients with IBD [7]. Elevated cytokines together with mood change and the chronic pain due to chronic constipation could lead to cognitive impairment of IBD patients. Although previous observational studies do not support that severity of symptoms had an impact on cognitive function in IBD patients [1]. Studies did not reach consistency and more clinical research are needed in the field of cognitive dysfunction of IBD patients.

Our study aims to identify the visceral obesity of IBD patients, evaluate its impact on chronic constipation, inflammation, immune function and cognitive function in the remission period of IBD.

## RESULTS

### Patients baseline characteristics

**Figure 1:**
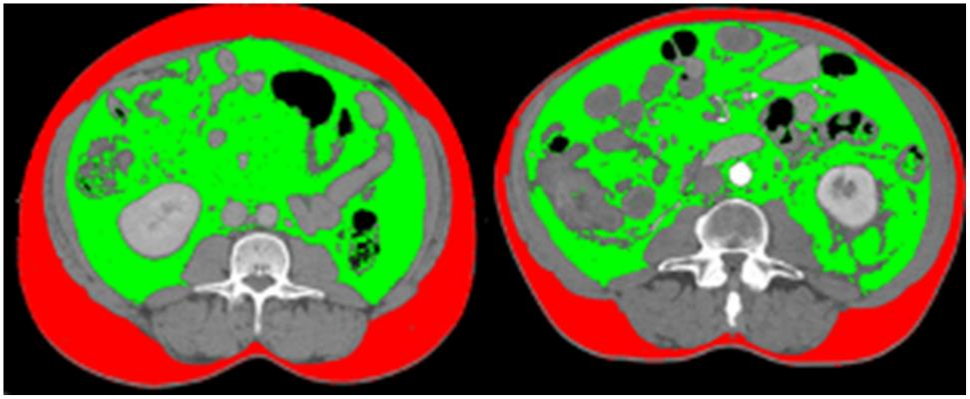
**Comparison of visceral obesity of two patients with same BMI.** The first patient has lower BMI than the other patient (20.1 kg/m^2^ vs. 29.3kg/m^2^), and its VAT/SAT ratio is also lower, which means that patient with lower BMI has less visceral obesity (VAT/SAT ratio: 1.22 vs. 2.48).

From July 2016 to January 2019, a total of 140 IBD patients were recruited. Characteristics of the IBD patients are shown in Table 1. The prevalence of visceral obesity was 38% for IBD patients (57 out of 150 patients), with 51% (37 out of 72) for CD patients and 26% for UC patients (20 out of 78 patients) respectively. The comparison of visceral obesity with non-visceral obesity patients with different BMI was illustrated in Figure 1 using sliceOmatic. In Figure 1, the first patient has lower BMI than the other patient, and its VAT/SAT ratio is also lower, which means that patient with lower BMI has less visceral obesity. The median age of our patients is 41 years old, with range of 22 to 68 years old. Females take a larger portion than males (72% *vs*. 28%). 72 patients were diagnosed with CD and 68 patients were diagnosed with UC. Patients had an average history of IBD for 7 years. 47% of these IBD patients admitted chronic constipation at recruitment. 23% of them had a history of treated anxiety or depression (Table 1). We analyzed the differences of disease duration, severity/category, and treatment between the visceral obesity group and non-visceral obesity group and found no statistical difference in these two groups (*P* > 0.05).

**Table 1:** Patients’ baseline characteristics.

**Characteristics**	**Total**
Age median, year (range)	41 (22-68)
Sex: female	108 (72%)
Sex: male	42 (28%)
Categories of disease (%)	
Crohn’s disease	78 (52%)
Ileal	35
Colonic	36
Ileocolonic	6
Pouch	1
Ulcerative colitis	72 (48%)
Isolated proctitis	32
Left-sided proctitis	31
Pancolitis	3
Pouch	6
Education level	
Middle school	27
High school	42
College	37
University or above	44
Chronic constipation	72 (48%)
Duration of disease, year (range)	7 (5-10)
History of treated anxiety/depression (%)	32 (21%)
Co-morbidities	
Diabetes	0
Cardiovascular diseases	0
Severity/category	
Mild	78 (52%)
Moderate	62 (41%)
Severe	10 (7%)
Treatment	
Corticosteroids	32 (21%)
Oral 5-aminosalicylates	96 (64%)
Infliximab	28 (19%)
Azathioprine	23 (15%)
Vedolizumab	8 (5%)

### Chronic constipation status

**Figure 2:**
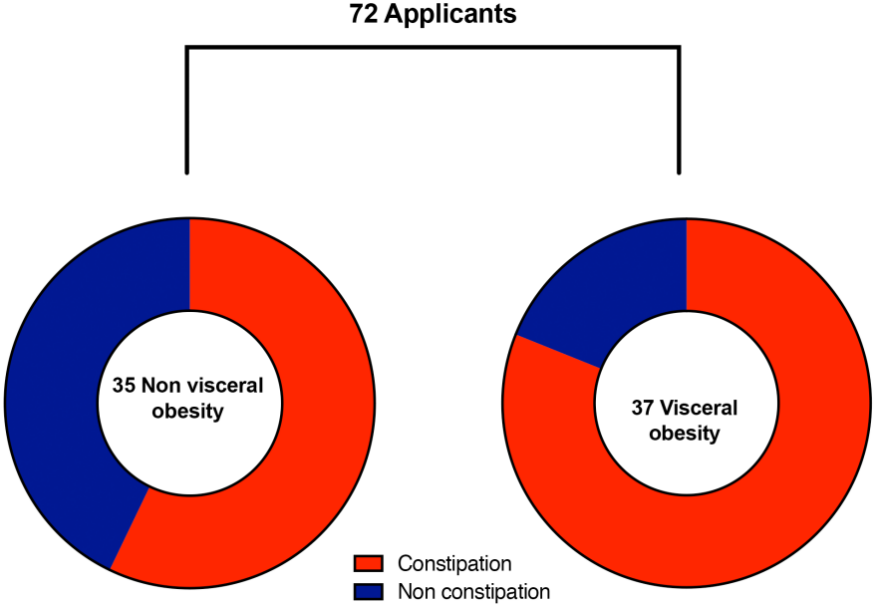
**Prevalence of chronic constipation between visceral obesity and non-visceral obesity patients with Crohn’s disease.** CD patients with visceral obesity suffered more from chronic constipation compared with CD patients without visceral obesity (81% vs. 57%, *P* = 0.028).

CD patients with visceral obesity has higher incidence of chronic constipation (81% vs. 57%, *P* = 0.028, Figure 2). However, for UC patients, the visceral obesity has no impact on chronic constipation (51% *vs*. 48%, *P* = 0.88).

### IL-6 level

**Figure 3:**
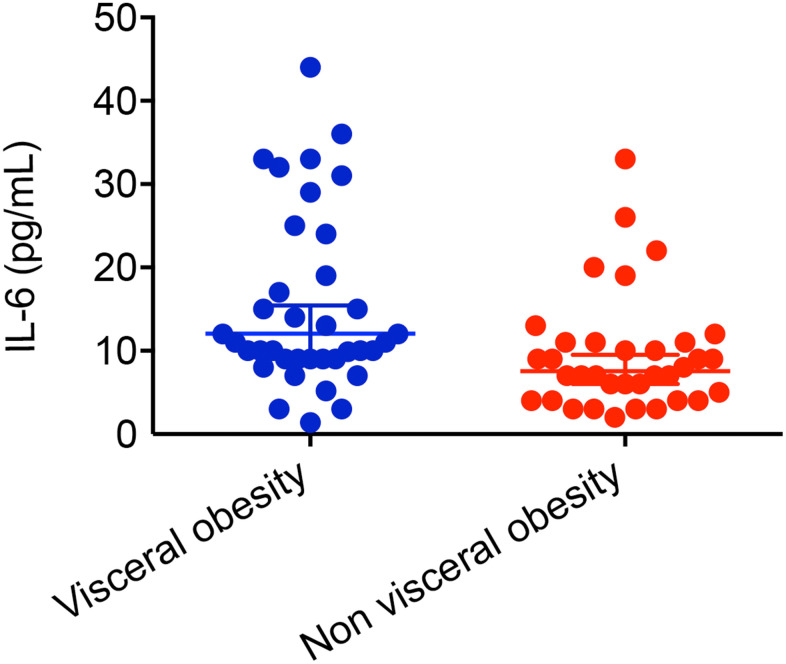
**Impact of visceral obesity on IL-6 in patients with Crohn’s disease.** CD patients with visceral obesity had higher level of IL-6 compared with CD patients without visceral obesity (15.28 ± 10.54 pg/ml, n=37 vs. 9.429 ± 6.94 pg/ml, n=35, *P* = 0.007).

**Figure 4:**
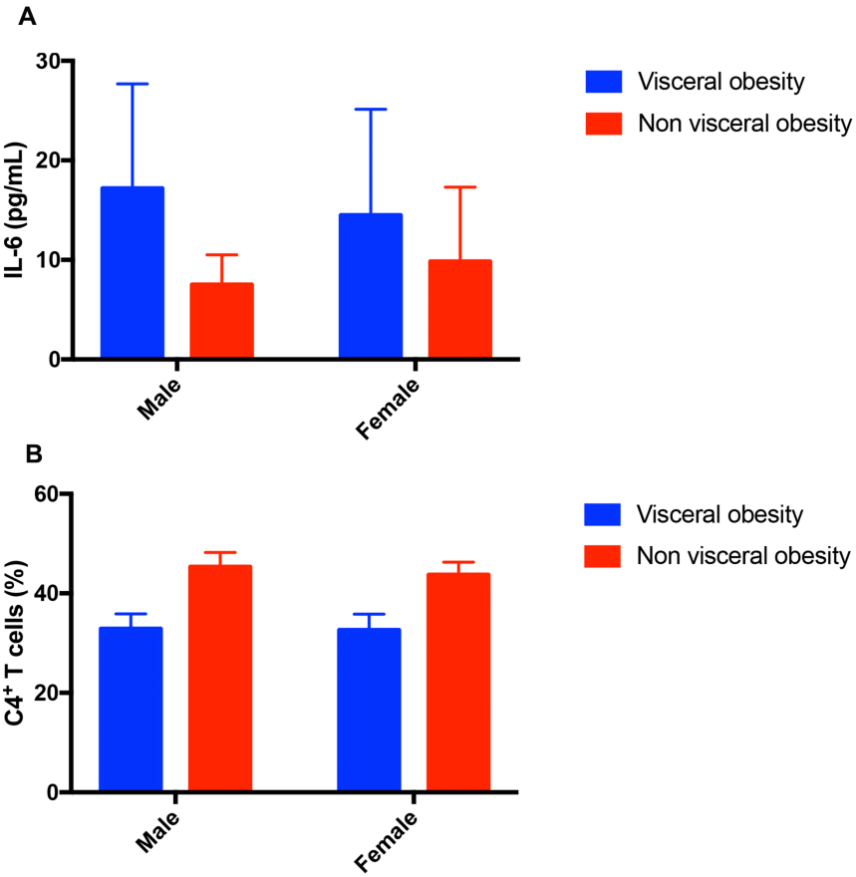
**Impact of visceral obesity in different sex groups in patients with Crohn’s disease.** (**A**) Impact of visceral obesity on IL-6. Male patients with visceral obesity had higher level of IL-6 compared with male patients without visceral obesity (17.18 ± 10.5 pg/ml, n=11 vs. 7.5 ± 3.02 pg/ml, n=6, *P* = 0.045). Female patients with visceral obesity tended to have higher level of IL-6 compared with female patients without visceral obesity (14.48 ± 10.66 pg/ml, n=26 vs. 9.83 ± 7.49 pg/ml, n=29, *P* = 0.065). (**B**) Impact of visceral obesity on CD4^+^ T cells. Male patients with visceral obesity had lower level of CD4^+^ T cells compared with male patients without visceral obesity (32.87 ± 3.03%, n=11 vs. 45.33 ± 2.88%, n=6, *P* <0.001). Female patients with visceral obesity had lower level of CD4^+^ T cells compared with female patients without visceral obesity (32.63 ± 3.2%, n=26 vs. 43.72 ± 2.56%, n=29, *P* <0.001).

CD patients with visceral obesity has higher IL-6 levels compared with CD patients without visceral obesity (15.28 ± 10.54 pg/ml, n=37 vs. 9.429 ± 6.94 pg/ml, n=35, *P* = 0.007, Figure 3). For patients with UC, patients with visceral obesity has the tendency of higher IL-6 levels (7.1 vs. 6.1 pg/ml, *P* = 0.058). Male patients with visceral obesity had higher level of IL-6 compared with male patients without visceral obesity (17.18 ± 10.5 pg/ml, n=11 vs. 7.5 ± 3.02 pg/ml, n=6, *P* = 0.045, Figure 4A). Female patients with visceral obesity tended to have higher level of IL-6 compared with female patients without visceral obesity (14.48 ± 10.66 pg/ml, n=26 vs. 9.83 ± 7.49 pg/ml, n=29, *P* = 0.065, Figure 4A).

### Immune function level

**Figure 5:**
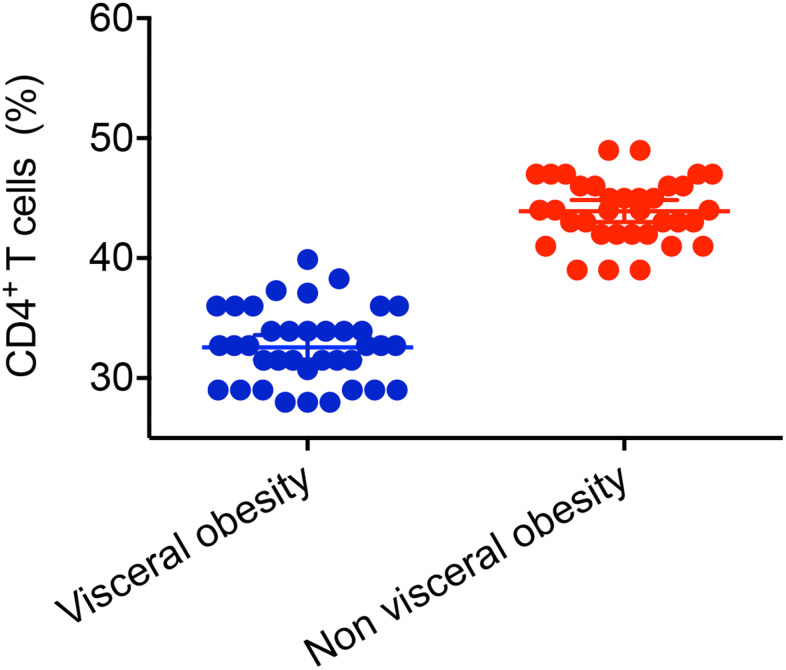
**Impact of visceral obesity on CD4^+^ T cells in patients with Crohn’s disease.** CD patients with visceral obesity had lower level of CD4^+^ T cells compared with CD patients without visceral obesity (32.7 ± 3.11%, n=37 *vs.* 44 ± 2.65%, n=35, *P* < 0.001).

CD patients with visceral obesity had lower CD4^+^ T cells (32.7 ± 3.11%, n=37 *vs.* 44 ± 2.65%, n=35, *P* < 0.001, Figure 5) compared with CD patients without visceral obesity. Male patients with visceral obesity had lower level of CD4^+^ T cells compared with male patients without visceral obesity (32.87 ± 3.03%, n=11 vs. 45.33 ± 2.88%, n=6, *P* <0.001, Figure 4B). Female patients with visceral obesity had lower level of CD4^+^ T cells compared with female patients without visceral obesity (32.63 ± 3.2%, n=26 vs. 43.72 ± 2.56%, n=29, *P* <0.001, Figure 4B). The impacts of visceral obesity on CD3^+^ T cells (70.9% *vs.* 69.1%, *P* = 0.347) and CD8^+^ T cells were not significant different (31.2% vs. 26.9%, *P* = 0.142). The impact of visceral obesity on UC patients’ CD3^+^ T cells, CD4^+^ T cells AND CD8^+^ T cells did not reach statistical significance.

### Association of VAT/SAT ratio with BMI

**Figure 6:**
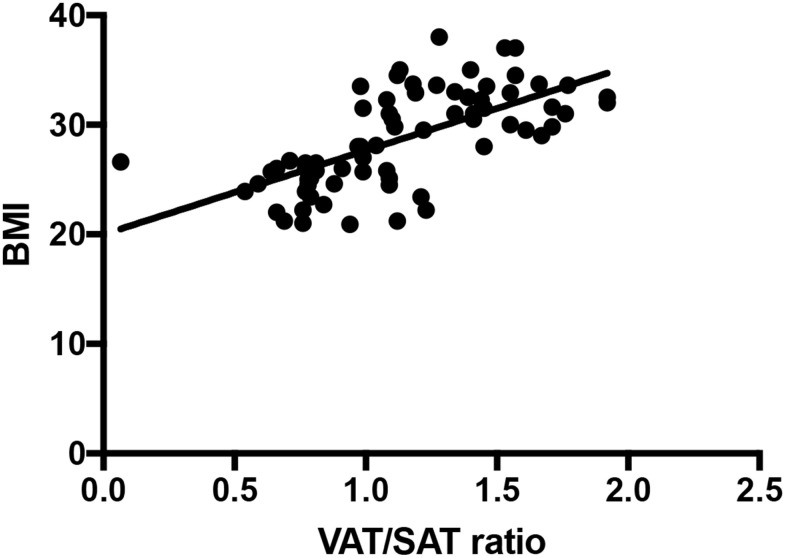
**VAT/SAT ratio correlates with BMI (r = 0.652, *P* < 0.001).**

The Person correlation r is 0.652 between VAT/SAT ratio and BMI (Figure 6, *P* < 0.001).

### Cognitive function

Levels of orientation to time, orientation to place, registration, attention and calculation, recall, language ability, repetition and complex commands were measured using the MMSE questionnaire. The prevalence of cognitive impairment for IBD in the remission period is relatively low. For 72 CD patients, only 9 patients had cognitive impairment with 5 patients with visceral obesity and 4 patients with non-visceral obesity. For UC patients, only 6 patients had cognitive impairment with 3 patients with visceral obesity and 3 patients with non-visceral obesity.

## DISCUSSION

Obesity has been a very important issue for IBD patients, especially in the remission period. High calories diet, sedentary behavior and lack of exercise all raise patients’ body weight and BMI. The chronic use of corticosteroids also could be the possible reasons for increased BMI and VAT content. The corticosteroid use was nearly twice as high for obese patients than for non-obese patients [13]. Cautions must be given to IBD patients in the remission period, otherwise their IBD may relapse and lead them into worse clinical outcomes. Actually previous study reported that due to their abdominal symptoms, their diet behaviors have changed a lot. 39% patients reported diet change. UC patients take higher amount of margarine, pasta and rice, and CD patients take more meat and cheese [14]. This eating behavior leads to on rising visceral obesity for IBD patients. More published articles pay attention only to BMI and reached various conclusions. A study proved that an association of increased risk of IBD patients with BMI > 40 kg/m^2^ with a seven-fold higher risk of postoperative infection [15]. Several studies have shown association of obesity with increased operative times, increased blood loss, and a higher risk of conversion from laparoscopic to open surgeries [16, 17]. In contrast to this, a retrospective cohort study including 391 IBD patients undergoing surgery found that 30-day postoperative complication rates including total complications, wound infection, or anastomotic leak did not vary according to BMI [18]. The inconsistency of these studies may because that they did not evaluate further into visceral obesity, which is a more representative parameter of body fat. VAT is not only a risk factor for the occurrence of gastrointestinal disorders but also can negatively impact clinical outcomes [19]. Our study suggested that VAT/SAT ratio is associated with BMI and CD patients with visceral obesity has higher chance of chronic constipation. A previous study of 958 Japanese adults who underwent colonoscopy suggested that BMI is a useful indicator for constipation [20]. In contrast with this study, we evaluated the association of VAT/SAT with chronic constipation and found positive association. What is more, constipation may due to various factors, such as a low fiber diet, painful defecation with stool withholding or probably due to slow gastrointestinal transit. More clinical studies are needed to prove our findings and interventions could be implemented for these visceral obesity CD patients.

Obese patients have higher levels of inflammatory microenvironment parameters, including C-reactive protein (CRP). Obese tissue is recognized as an endocrine organ. The adipose tissue compartments are infiltrated by inflamed macrophages, which release inflammatory parameters [21]. IL-6 can be used as an inflammatory marker for severe infection. IL-6 stimulates the inflammatory and auto-immune process in many diseases such as diabetes, cancer and Alzheimer’s disease. Moreover, T-cells from adipose tissue adjacent to inflamed segments of the intestine of CD patients produce more IL-6 [22]. Our study proved was the first to evaluate the association of visceral obesity with IL-6 and immune function for IBD patients in the remission period. Visceral obesity increased IL-6 and CD4^+^ T cells for CD patients. UC patients with visceral obesity also has the tendency to develop higher level of IL-6, indicating the role of visceral obesity on chronic inflammation and decreased immune function for IBD patients. In our study, only CD patients with visceral obesity suffered from chronic constipation, increased IL-6 level, and decreased immune function rather than UC patients. That might due to the rather higher prevalence of visceral obesity in CD patients in comparison to UC patients (51% vs. 26%). For UC patients, patients with visceral obesity have the tendency of higher IL-6 levels (7.1 vs. 6.1 pg/ml, *P* = 0.058). But the difference did not reach statistical significance. Thus, further studies with larger sample size are needed to validate our results.

Meta-analysis has shown strong association of between obesity and Alzheimer's disease (AD), and other dementias [23, 24]. Obesity increases the risk of development into AD in geriatric patients. The cognitive impairment in IBD patients might due to their change of microbiome. There is growing evidence that gut microbiota could modulate brain functions and consequent behavior through gut-brain axis (GBA) [25]. Lack of normal gut flora may have a significant impact on adult cognition and their react to sad mood [26]. Previous observational studies in patients with irritable bowel syndrome and IBD proved that, IBD patients seems not to have a statistically significant cognitive impairment. It has been hypothesized that IBD patients with mood disorders may affect the cognitive performance of query machine specific tasks. Our observational study do not support the hypothesis that visceral obesity has statistically significant impact on cognitive function, neither for CD patients nor for UC patients. Our study reached the conclusion that IBD patients with visceral obesity have no impact on cognitive impairment. The IBD patients in the remission period did not develop a high prevalence of cognitive impairment.

We admit that our study has several limitations. Our study is an cross-sectional observational study. We did not record patients’ diet habits or sedentary behavior, which may impact patients’ visceral obesity. We did not evaluate patients’ hormone levels, such as leptin and insulin, sex hormones and growth hormone that could influence their appetite and thus have impact on their VAT/SAT ratio. However, our study is the first to take visceral obesity into consideration for IBD patients in the remission period. It worth further studies in the field and more interventions are needed to help patients control their visceral obesity. It is a problem that could be reverse and more interventions definitely would lead to better control of the disease.

## CONCLUSIONS

In conclusion, visceral obesity lead to chronic constipation, higher level of IL-6 and lower immune function for CD patients in the remission period. No cognitive impairment was found to be associated with visceral obesity.

## MATERIALS AND METHODS

### Study design and patients

In our observational study, all patients were aged between 22 to 68 year with at least five years of IBD history and now in the remission period of IBD July 2016 to January 2019. Remission for UC patients mean complete cessation of rectal bleeding, urgency and increased stool frequency. Remission for CD patients mean that patients’ inflammation stop causing painful damage to patients colon and rectum. The diagnose of IBD was checked in the medical record of all recruited patients. All patients were diagnosed using a combination of endoscopy (for CD) or colonoscopy (for UC) and imaging technologies, such as magnetic resonance imaging (MRI) scans or CT scans. Stool samples were checked to guarantee that all clinical symptoms were not caused by an infection. Blood tests were run sometimes in patients’ history to help confirm the IBD. Patients previous diagnosis of cognitive impairment was recorded if they had a history of treated anxiety or depression. 140 patients with IBD were recruited from department of anorectal surgery at affiliated hospital of Nanjing University of Chinese Medicine, Jiangsu Province Hospital of Chinese Medicine between July 2016 and January 2019. Patients’ abdominal CT scans, baseline clinical characteristics including chronic constipation history, patients’ education status, IL-6 level, immune cells including the following cell groups: CD3^+^ T cells, CD4^+^ T cells as well as CD8^+^ T cells, MMSE questionnaire were taken at recruitment.

### Visceral obesity evaluation

Visceral adipose tissue (VAT) and subcutaneous adipose tissue (SAT) (square centimeters) were measured with SliceOmatic software (version 5.0, Tomovision, Magog, Quebec, Canada) using abdominal CT scans at recruitment. Based on previous publications, we chose the third lumbar spine (L3) as the standard delimiter because L3 levels appear to be the most relevant and recognized delimiter for whole body adipose tissue [27]. The structures of VAT and SAT were quantified based on pre-established thresholds of Hounsfield units (HU) ranged from -150 to -50 HU for VAT and -190 to -30 HU for SAT according to previously published articles [28, 29]. Visceral obesity was defined using VAT/SAT ratio for males > 1.33 and for females > 0.93 as previously described [30].

### IL-6 evaluation

All serum samples were collected at the time of patients recruitment. Measurement of serum level of cytokine IL-6 was carried out using enzyme-linked immunosorbent assay (ELISA) Kit (Zhongkang Biotech, Hangzhou, China) following manufacturer’s instructions.

### Flow cytometry

Cells were collected after incubation time, then the cells were washed in PBS and submitted to flow cytometry to determine the proportion of T cells using antibodies from Beckman Coulter: anti-CD3, anti-CD4 and anti-CD8. Cells were incubated for 20 minutes at 4 degree Celsius in the darkness, after that cells were washed by centrifugation and acquired in flow cytometer within 24 hours. Single stained controls were used to set compensation parameters. After the acquisition, flow cytometric analyses using FlowJo v7.6.1 software to evaluate the frequencies of CD3^+^ T cells, CD4^+^ T cells as well as CD8^+^ T cells.

### Evaluation of cognitive function analysis

For all of the recruited IBD patients, MMSE evaluation was collected at the recruitment time. The MMSE is a 30-point questionnaire that is widely used in clinical and research settings to define if a patients has cognitive impairment with high sensitivity of 80%-90% and also high specificity of 70%-80%. It has the advantage of being highly sensitive and easy to operate. MMSE contains 8 categories, including (1) the orientation ability to time, (2) orientation ability to place, (3) registration ability, (4) attention and calculation ability, (5) recall ability, (6) language ability, (7) repetition and (8) complex commands ability. MMSE score is the total score of the above 8 categories. Higher scores indicate that this patient has a better cognitive function. Cognitive impairment was defined as according the educational levels of patient, for illiterates cognitive impairment was defined ≤14, for patients with elementary school cognitive impairment was defined as ≤17, and for patients with over elementary school education cognitive impairment was defined as ≤22 according to previous published definitions [31].

### Statistics

Descriptive statistical analyses of baseline characteristics were conducted for all continuous variables by mean values (percentage) and categorical variables by numbers (percentage). Comparisons of IL-6 levels and immune cells between visceral obesity and non-visceral obesity subgroups were made using ANOVA. Categorical data like prevalence of chronic constipation, is presented with absolute numbers and percentages, and was analyzed using Chi-squared tests. Results of the cognitive impairment from MMSE questionnaire were initially compared between groups using ANOVA. We also draw a scatter plot to show the tendency of association of VAT/SAT ratio with BMI. A linear regression was applied to quantify the strength of the relationship between these two parameters. All analysis was performed using SPSS, version 16.0 (IBM Corporation, Armonk, NY, USA).

### Ethics approval and consent to participate

The studies involving human subjects were reviewed and approved by the Medical Ethics Review Committee of affiliated Hospital of Nanjing University of Chinese Medicine, Jiangsu Province Hospital of Chinese Medicine. All patients who participated in this study provided written informed consent during the study.

### Availability of data and material

All data and material are available from the corresponding author on reasonable request.
